# COVID‐19, HIV and key populations: cross‐cutting issues and the need for population‐specific responses

**DOI:** 10.1002/jia2.25632

**Published:** 2020-10-01

**Authors:** Jenny Iversen, Keith Sabin, Judy Chang, Ruth Morgan Thomas, Garrett Prestage, Steffanie A Strathdee, Lisa Maher

**Affiliations:** ^1^ Kirby Institute for Infection and Immunity Faculty of Medicine UNSW Sydney Sydney NSW Australia; ^2^ Joint United Nations Programme on HIV/AIDS (UNAIDS) Geneva Switzerland; ^3^ International Network of People who use Drugs London United Kingdom; ^4^ Global Network of Sex Work Projects Edinburgh United Kingdom; ^5^ Global Health Sciences Department of Medicine University of California San Diego La Jolla CA USA

**Keywords:** COVID‐19, HIV, key populations, physical distancing, vulnerability, health equity

## Abstract

**Introduction:**

Key populations at elevated risk to contract or transmit HIV may also be at higher risk of COVID‐19 complications and adverse outcomes associated with public health prevention measures. However, the conditions faced by specific populations vary according to social, structural and environmental factors, including stigma and discrimination, criminalization, social and economic safety nets and the local epidemiology of HIV and COVID‐19, which determine risk of exposure and vulnerability to adverse health outcomes, as well as the ability to comply with measures such as physical distancing. This commentary identifies common vulnerabilities and cross‐cutting themes in terms of the impacts of COVID‐19 on key populations before addressing issues and concerns specific to particular populations.

**Discussion:**

Cross‐cutting themes include direct impacts such as disrupted access to essential medicines, commodities and services such as anti‐retroviral treatment, HIV pre‐exposure prophylaxis, opioid agonist treatment, viral load monitoring, HIV and sexually transmitted infections testing, condoms and syringes. Indirect impacts include significant collateral damage arising from prevention measures which restrict human rights, increase or impose criminal penalties, and expand police powers to target vulnerable and criminalized populations. Significant heterogeneity in the COVID‐19 pandemic, the underlying HIV epidemic and the ability of key populations to protect themselves means that people who inject drugs and sex workers face particular challenges, including indirect impacts as a result of police targeting, loss of income and sometimes both. Geographical variations mean that transgender people and men who have sex with men in regions like Africa and the middle east remain criminalized, as well as stigmatized and discriminated against, increasing their vulnerability to adverse outcomes in relation to COVID‐19.

**Conclusions:**

Disruptions to both licit and illicit supply chains, loss of income and livelihoods and changes in behaviour as a result of lockdowns and physical distancing have the potential to exacerbate the impacts of the COVID‐19 pandemic on key populations. While these impacts will vary significantly, human‐rights approaches to COVID‐19 emergency laws and public health prevention measures that are population‐specific and sensitive, will be key to reducing adverse health outcomes and ensuring that no one is left behind.

## 1 INTRODUCTION

The rapid spread of coronavirus disease (COVID‐19) has induced governments worldwide to introduce preventive measures, including physical distancing, bans on public gatherings, workplace and school closures and lockdowns designed to reduce contact and suppress transmission of Severe Acute Respiratory Syndrome Coronavirus 2 (SARS‐Cov2), the virus that causes COVID‐19. In the absence of a vaccine or effective pharmaceutical treatments, modelling indicates the potential of non‐pharmaceutical interventions to reduce COVID‐19‐related demand on health systems by two‐thirds and to halve deaths [1].

People who inject drugs (PWID), sex workers, men who have sex with men (MSM), transgender people, and people in prisons and closed settings, comprise key populations in the response to HIV [2]. In 2020, key populations and their sexual partners accounted for almost two‐thirds of new HIV infections globally [3]. Older people, men, and those with medical comorbidities including chronic pulmonary disease, cardiovascular disease, cerebrovascular disease, diabetes and compromised immunity, are at higher risk of COVID‐19 complications [4]. Several risk factors for complications, including smoking and vaping drugs, are elevated among key populations [5]. In many settings, key populations also face stigma and discrimination, criminalization, homelessness and food insecurity, which may also exacerbate vulnerability to COVID‐19 complications. However, conditions faced by key populations vary according to social, structural and environmental factors, including the underlying epidemiology of both HIV and COVID‐19. As highlighted by Sohn *et al*. [6], the concentration of COVID‐19 risk is similar to HIV, where overlapping and intersecting individual, network and structural risks influence both acquisition and transmission and vulnerability to adverse health outcomes [7], as well as the ability to comply with public health prevention measures.

This commentary identifies common vulnerabilities and cross‐cutting themes including access to prevention, treatment and care, and the need to respect health and human rights during the COVID‐19 pandemic, before addressing population‐specific issues and concerns.

## 2 DISCUSSION

### 2.1 Common vulnerabilities and cross‐cutting themes

Trust and respect for human rights have long been recognized as central to effective responses to HIV. Four decades of the HIV epidemic have taught us that restrictive and stigmatizing measures drive people underground, perpetuate stigma, erode trust and respect for human rights and disproportionately impact vulnerable populations [8]. The potential for both the COVID‐19 pandemic, and the responses to it, to amplify existing inequalities underlines the need to address criminalization, stigma and discrimination, which are also structural drivers of the HIV epidemic. Discriminatory law enforcement and overly restrictive lockdown orders may disproportionately impact key populations and undermine public health strategies and community trust in Government [8] with increasing reports of sex workers, PWID, MSM and transgender people being fined arrested or detained for breaching COVID‐19 related restrictions [9].

It is important to remember that the HIV epidemic is not over, with 38 million people living with HIV (PLHIV) globally and 1.7 million new infections in 2019 [3]. COVID‐19‐induced supply chain issues may also disproportionately impact key populations reliant on access to medications such as anti‐retroviral treatment (ART), pre‐exposure prophylaxis (PrEP) and opioid agonist treatment (OAT), as well as services such as viral load monitoring, HIV and STI testing and condom and needle syringe distribution. In July 2020, the World Health Organization (WHO) reported that numerous countries had experienced disruptions to provision of ART during the COVID pandemic [10]. Clinics around the world have reduced hours, reallocated staff or closed, leaving millions of PLHIV with uncertain access to treatment [10]. South Africa, with 20% of the world’s PLHIV and 3.5 of an estimated 7.7 million PLHIV not virally suppressed [11], has the highest COVID‐19 cases in Africa and ranks in the top 20 globally for COVID‐19 deaths per 100,000 population [12].

In addition to disruptions in access to essential medicines, commodities and health services, some key populations are at increased risk of indirect impacts arising from responses to COVID‐19, particularly physical distancing measures. The negative consequences of these measures on general population health and well‐being, such as mental health issues arising from isolation, loss of income and residential instability, will be exacerbated in vulnerable key populations who lack the resources to physically distance or who do not have access to social safety nets or the option of working from home [6]. In low‐income countries characterized by a high burden of infectious diseases, including tuberculosis, cholera, typhoid, malaria and HIV/AIDS, structural and environmental conditions which impede adherence to physical distancing and hygiene measures will differentially impact key populations [13].

UNAIDS has cautioned against the use of COVID‐19 emergency powers or public health justifications to restrict human rights and expand police powers to target vulnerable and criminalized groups [14, 15] and the United Nations Office of the High Commissioner for Human Rights (OHCHR) has expressed concerns about the use of imprisonment for non‐compliance with public health measures [16]. There is a need for independent mechanisms to oversee the use of police powers and to ensure that police are accountable for their actions during the pandemic. To safeguard against the pandemic being used to introduce or expand laws, penalties and police powers that criminalize key populations, UNAIDS recommends that COVID‐19 emergency laws and powers are necessary, proportionate, non‐arbitrary, evidence‐informed and lawful, as well as time‐limited and renewable only through appropriate democratic mechanisms [17]. Across all key populations, meaningful community participation by civil society will be essential to minimize the potential for collateral damage, maintain momentum towards Global HIV targets and to ensure that the COVID‐19 response, or “cure,” is not worse than the disease itself.

### 2.2 Population‐specific challenges

Global networks, including the International Network of People who use Drugs (INPUD), the Global Network of Sex Work Projects (NSWP), the Global Network of People Living with HIV (GNP+) and MPact Global Action for Gay Men’s Health and Rights have issued statements calling for urgent action to protect their communities and to address population‐specific needs for prevention, care and treatment [9, 18, 19, 20]. Challenges faced by key populations and how networks and communities are responding vary; below we highlight some of these considerations and contexts in order to illustrate the heterogeneity of the COVID‐19 pandemic and its impacts.

#### 2.2.1 People who inject drugs

Compared to the general population, PWID have a high burden of comorbid medical conditions [21], exacerbated by criminalization and socio‐economic disadvantage [22], which place them at greater risk of infection and complications. Restrictions on access to key services for PWID have the potential to increase overdose deaths and HIV and HCV transmission, undermining gains made by global elimination efforts [23]. These services, including Needle Syringe Programs (NSP) and OAT, have historically required frequent attendance and restricted access to takeaway OAT [24]. COVID‐19 travel bans and border closures are also impacting illicit markets, with shortages of precursor chemicals, declining availability of opioids and increasing prices in some settings [25] potentially increasing demand for OAT and naloxone [24].

Countries such as Nepal and Morocco, with moderate HIV prevalence among PWID at 7 to 9% and limited COVID‐19 epidemics with less than one death per 100,000 population [12], have responded by working to ensure supplies of OAT for multimonth dispensing (MMD) and providing unsupervised dosing. Canada, a country with 11% HIV prevalence among PWID, and a severe COVID‐19 epidemic with 21.3 deaths per 100,000 population [12], has introduced biometric vending machines that dispense prescribed supplies of hydromorphone tablets to registered patients [26]. As government services have closed or reduced their hours, drug users and peer‐run services, have stepped up to maintain and provide harm reduction services to PWID, as well as distributing OAT, ART and HCV medications to those in lockdown [27]. Modifications have also been made to harm reduction programmes, including the distribution of supplies through outreach, pharmacies, vending machines and post and services previously rejected or under‐scaled by policy makers, such as take‐home dosing of methadone or hydromorphone. While policy changes to accommodate unsupervised OAT demonstrate not only that flexibility in OAT delivery is possible and can be done safely and effectively, OAT continues to be unavailable in many settings including Bahrain, Belarus, Brazil, Cameroon, Egypt, Nigeria and Russia [27].

#### 2.2.2 Sex workers

In many settings sex workers remain vulnerable to HIV and other sexually transmitted infections (STI) due to multiple factors including criminalization, challenges negotiating consistent condom use, unsafe working environments, stigmatization, discrimination and violence [28, 29]. Global COVID‐19 induced bans on sex work, including closures of brothels, mean that sex workers have faced loss of income and are unable to provide for themselves and their families [18]. Furthermore, reduced numbers of clients and school closures may disproportionately impact women with responsibility for school‐aged children. In most countries, national social protection schemes and emergency protection measures put in place for workers exclude sex workers, particularly where sex work is criminalized. This exclusion means that many sex workers are faced with putting their safety, health and lives at increased risk in order to survive. Sex worker organizations have responded rapidly by providing resources, including getting started in online/non‐contact work, working safely during the COVID‐19 pandemic and dealing with stress and emotional impacts, as well as implementing income support programmes. Bangladesh, where HIV prevalence among sex workers is low at 0.2% and COVID‐19 related deaths are also low at less than one per 100 000 population [12], is one of the few countries that has provided emergency income support for sex workers. In the absence of income support, sex work may be driven further underground with significant health and safety risks [30]. Migrant sex workers and those who use drugs may be particularly vulnerable to exploitation by clients, including unsafe work practices and lower prices. Sex workers have been targeted by police for physical distancing offences in several countries and COVID‐19‐related policing of public health, including punitive crackdowns, raiding of homes, compulsory testing, arrests and threatened deportations of migrant sex workers [18], has the potential to undermine access to health services, as well as sex workers’ ability to report crimes against them.

#### 2.2.3 Men who have sex with men

Social connectedness has been shown to promote health seeking and risk reduction, including access to HIV treatment and HIV PrEP among gay and bisexual men (GBM) [31]. Lockdowns and physical distancing also threaten to undermine the centrality of peer support to optimizing health outcomes in this population. However, early research suggests that some GBM in high‐income countries have adapted their sexual behaviour [32]. In Australia where HIV prevalence in this population is 18% and COVID‐19 transmission has been limited to date, GBM dramatically reduced their sexual contacts following the introduction of physical distancing restrictions [33]. For many in the Lesbian, Gay, Bisexual, Transgender and Intersex (LGBTI) community, the family home may not be a safe place. In Uganda where HIV prevalence among GBM is estimated at 85%, a raid on an LGBTI community shelter resulted in 19 people arrested and detained without access to bail, legal representation or medication for allegedly violating physical distancing measures [14, 20]. Their release was eventually secured after significant efforts by civil society and a court later awarded compensation for rights violations [8].

#### 2.2.4 Prisoners and detainees

On any given day, <10 million people, including pre‐trial detainees, are incarcerated worldwide, with an estimated 3.8% living with HIV, 15.1% with HCV, 4.8% with chronic HBV and 2.8% with active tuberculosis (TB) [17], in conditions where physical distancing is impossible. Many more are detained in compulsory drug detention, asylum seeker and immigration detention, and private drug treatment centres [9]. In the US, COVID‐19 outbreaks have been reported in prisons and jails, including in New York, Illinois and Ohio, with both staff and detainees infected [34, 35]. Interim guidance by the United Nations OHCHR and the WHO has urged governments to reduce the number of people in detention by finding ways to release those at increased risk of COVID‐19, including older detainees and people with underlying health conditions, as well as children and those with low risk profiles and people incarcerated for minor offences [16].

#### 2.2.5 Transgender people

Estimates of the size of the transgender population vary by location, however population‐based surveys report 0.5% to 1.0% of adults identify with a gender different to their sex assigned at birth [36]. Similarly, HIV prevalence estimates among transgender populations are highly variable within and across geographic locations, although evidence suggests transgender populations are disproportionately impacted by HIV [37]. In some Latin American countries, where HIV prevalence among transgender feminine people is typically >10% [36], Governments implemented gender‐based lockdown policies, with designated days residents were permitted to leave the home based on their gender. These policies led to reports of discrimination and violence against transgender people who were away from home on a day that corresponded to their gender identity but did not match the gender listed on their identification documents [8].

In many settings, disruptions to both licit and illicit supply chains, loss of livelihoods, changes in behaviour as a result of lockdowns and physical distancing and discriminatory and coercive policing have the potential to inflict more damage on vulnerable communities than SARS‐Cov2. UNAIDS and WHO have called for a “people‐centred approach” to ensure access to medication is maintained throughout the COVID‐19 pandemic [17]. Expediting differentiated service delivery such as telehealth and expanded MMD for ART for PLHIV and OAT and NSP for PWID [38], and the roll‐out of adaptive programmes like social protection support for sex workers, are essential to ensure that COVID‐19 is not used to disregard and further disenfranchise key populations. Communities are uniting to find solutions and several countries, including African and South American nations, have developed and/or implemented community‐based ART distribution policies to reduce demands on health systems and to encourage people to stay at home [8].

COVID‐19 has the potential to reverse decreases in HIV, TB and viral hepatitis mortality, however, its impacts on key populations are likely to be uneven. PWID and sex workers face particular challenges in relation to physical distancing, including indirect impacts as a result of police targeting, loss of income and sometimes both. Geographical variations mean that MSM and transgender people in regions like Africa and the middle east remain criminalized, as well as stigmatized and discriminated against, increasing their risk of adverse outcomes. While successful containment of SARS‐CoV‐2 in community settings also protects prisoners and detainees, this group remains vulnerable to the negative health consequences of social isolation. And under COVID‐19 pandemic conditions, PLHIV in resource constrained settings with fragile health systems will be more likely than PLHIV in the global north to experience ART interruptions which compromise their health.

Research is also needed to guide public health responses. Consistent with the “right to science”’ [39], interventions to prevent, diagnose and treat COVID‐19 need to be accessible and available to all, especially key populations for whom COVID‐19 is a pandemic on top of one or more epidemics. This has been reinforced by a recent call for a people’s vaccine which guarantees COVID‐19 vaccines are available free of charge to everyone, everywhere with access prioritized for front‐line workers, vulnerable people and low‐ and middle‐income countries [40]. Biomedical, socio‐economic and behavioural data on the impacts of COVID‐19 on key populations are needed, including studies designed to assess how specific policy responses increase or decrease exposure to harmful consequences and to monitor the impact of changes to service delivery. Existing surveillance mechanisms, with appropriate rights‐based legal safeguards, must be adapted to assess the impacts of COVID‐19 on established epidemics, including HIV and viral hepatitis, in key populations [32]. Understanding the impacts of lockdowns and physical distancing measures on key populations will be critical to monitoring trends in HIV and other infections.

## 3 CONCLUSIONS

Heterogeneity in the COVID‐19 pandemic and in the ability of key populations to protect themselves from COVID‐19 and its consequences necessitates rapid development and implementation of evidence‐informed interventions that address the population determinants of transmission and local risks, while remaining sensitive to differences in the needs of key populations and the synergistic impacts of structural factors on particular communities. One of the key lessons from the HIV epidemic was that prevention responses are more effective when communities are empowered with knowledge about the virus and how to mitigate risk and are involved in, or lead, the process of developing inclusive responses. Greater solidarity with, more guidance from, and the meaningful involvement of, key populations who are working to fill gaps in prevention, care and treatment, information and advocacy, is required to shape the COVID‐19 response. While COVID‐19 has exposed the moral and political barriers to implementing evidence‐based public health responses [41], the willingness to set aside ideological objections to services such as unsupervised OAT in order to save lives may be short‐lived. There is an urgent need for advocacy to ensure that the introduction or scale up of evidence‐based interventions during COVID‐19 are sustained and integrated into routine service delivery. Working together to understand how key populations experience, engage with and emerge from, COVID‐19 pandemic‐induced change, developing responses that address population‐specific needs, and ensuring that no‐one is left behind, will be vital to a post‐COVID‐19 transition to a more sustainable, equitable and resilient society.

## COMPETING INTERESTS

No competing interests declared.

## AUTHORS’ CONTRIBUTIONS

JI, KS, JC, RMT, GP, SS and LM collectively identified the need for this work and contributed ideas and perspectives. JI and LM prepared the first draft with input from KS and SS. All authors provided critical feedback and helped to shape the analysis and revise successive drafts of the manuscript.

## ABBREVIATIONS

ART, Anti‐Retroviral Treatment; COVID‐19, Coronavirus Disease; GBM, Gay and bisexual men; GNP+, Global Network of People Living with HIV; INPUD, International Network of People who use Drugs; LGBTI, Lesbian, Gay, Bisexual, Transgender and Intersex; MMD, Multi‐Month Dispensing; MSM, Men who have Sex with Men; NSP, Needle Syringe Programs; NSWP, Global Network of Sex Work Projects; OAT, Opioid Agonist Treatment; OHCHR, United Nations Office of the High Commissioner for Human Rights; PLHIV, People Living with HIV; PrEP, Pre‐exposure Prophylaxis; PWID, People who Inject Drugs; SARS‐Cov2, Severe Acute Respiratory Syndrome Coronavirus 2; STI, Sexually Transmitted Infections; TB, Tuberculosis; UNAIDS, Joint United Nations Programme on HIV/AIDS; WHO, World Health Organization.

## References

[1] Ferguson N , Laydon D , Nedjati Gilani G , Imai N , Ainslie K , Baguelin M , et al.Report 9: Impact of non‐pharmaceutical interventions (NPIs) to reduce COVID19 mortality and healthcare demand. Imperial College COVID‐19 Response Team 16 March 2020 [cited 2020 Sep 10]. Available from: https://spiral.imperial.ac.uk:8443/handle/10044/1/77482

[2] World Health Organization . Consolidated Guidelines on HIV prevention, diagnosis, treatment and care for key populations. Geneva: 2014 [cited 2020 Apr 8]. Available from: https://www.who.int/hiv/pub/guidelines/keypopulations/en/ 25996019

[3] Joint United Nations Programme on HIV/AIDS (UNAIDS) . UNAIDS Data 2020. Geneva: UNAIDS [cited 2020 Sep 10]. Available from: https://www.aidsdatahub.org/sites/default/files/resource/unaids‐2020‐aids‐data‐book.pdf

[4] US Centers for Disease Control (CDC) . COVID‐19: Groups at Higher Risk for Severe Illness [cited 2020 Apr 23]. Available from: https://www.cdc.gov/coronavirus/2019‐ncov/need‐extra‐precautions/groups‐at‐higher‐risk.html

[5] Verster A , MacDonald V , Luhman N .WHO guidance on COVID‐19 relevant to key populations [cited 2020 Apr 23]. Available from: https://www.who.int/docs/default‐source/searo/thailand/who‐guidance‐on‐covid‐19‐relevant‐to‐kp‐080420.pdf?sfvrsn=d2cfacba_0

[6] Sohn AH , Phanuphak N , Baral S , Kamarulzaman A . Know your epidemic, know your response: understanding and responding to the heterogeneity of the COVID‐19 epidemics across Southeast Asia. J Int AIDS Soc. 2020;23:e25557. 32623817 10.1002/jia2.25557 PMC7300799

[7] Global Commission on HIV and the Law . Global Commission on HIV and the Law: risks, rights & health. Supplement. New York: UNDP; 2018 [cited 2020 Apr 8]. Available from: https://hivlawcommission.org/wp‐content/uploads/2019/11/Hiv‐and‐the‐Law‐supplement_EN.pdf

[8] UNAIDS . Rights in a pandemic – Lockdowns, rights and lessons from HIV in the early response to COVID‐19 [cited 2020 Sep 10]. Available from: https://www.unaids.org/sites/default/files/media_asset/human‐rights‐and‐covid‐19_en.pdf

[9] International Network of People who use Drugs (INPUD) . In the time of COVID‐19: Civil Society Statement on COVID‐19 and People who use Drugs [cited 2020 Apr 8]. Available from: https://www.inpud.net/en/time‐covid‐19‐civil‐society‐statement‐covid‐19‐and‐people‐who‐use‐drugs

[10] Doherty M .What we know about HIV and COVID‐19. Proceedings of AIDS 2020: Virtual, July 6–8 [cited 2020 Sep 15]. Available from: https://cattendee.abstractsonline.com/meeting/9289/presentation/952

[11] Drain PK , Garrett N . SARS‐CoV‐2 pandemic expanding in sub‐Saharan Africa: Considerations for COVID‐19 in people living with HIV. Lancet. 2020;22:100342. 10.1016/j.eclinm.2020.100342 PMC7174187 32322805

[12] John Hopkins University . Mortality analysis: How does mortality differ across countries (10 June 2020). 2020 [cited 2020 Jun 10]. Available from: https://coronavirus.jhu.edu/data/mortality

[13] Pūras D , de Mesquita JB , Cabal L , Maleche A , Meier BM . The right to health must guide responses to COVID‐19. Lancet. 395(10241):1888‐90. 10.1016/S0140-6736(20)31255-1 PMC7259895 32479828

[14] UNAIDS . UNAIDS condemns misuse and abuse of emergency powers to target marginalized and vulnerable populations [cited 2020 Apr 23]. Available from: https://www.unaids.org/en/resources/presscentre/pressreleaseandstatementarchive/2020/april/20200409_laws‐covid19

[15] United Nations Office of the High Commissioner on Human Rights (OHCHR) . COVID‐19: States should not abuse emergency measures to suppress human rights – UN experts [cited 2020 Apr 23]. Available from: https://www.ohchr.org/EN/NewsEvents/Pages/DisplayNews.aspx?NewsID=25722

[16] United Nations Office of the High Commissioner for Human Rights (OHCHR) and WHO . Interim guidance COVID‐19: Focus on persons deprived of their liberty [cited 2020 Apr 23]. Available from: https://interagencystandingcommittee.org/system/files/2020‐03/IASC%20Interim%20Guidance%20on%20COVID‐19%20‐%20Focus%20on%20Persons%20Deprived%20of%20Their%20Liberty.pdf

[17] UNAIDS . Rights in the time of COVID‐19 Lessons from HIV for an effective, community‐led response [cited 2020 Apr 23]. Available from: https://www.unaids.org/en/resources/documents/2020/human‐rights‐and‐covid‐19

[18] Global Network of Sex Work Projects (NSWP) and UNAIDS . Sex workers must not be left behind in the response to COVID‐19 [cited 2020 Apr 8]. Available from: https://www.unaids.org/en/resources/presscentre/pressreleaseandstatementarchive/2020/april/20200408_sex‐workers‐covid‐19

[19] GNP+ . Statement on COVID‐19 criminalisation [cited 2020 Apr 8]. Available from: https://www.gnpplus.net/statement‐on‐covid‐19‐criminalisation/

[20] UNAIDS and MPact are extremely concerned about reports that LGBTI people are being blamed and abused during the COVID‐19 outbreak [cited 2020 May 6]. Available from: https://mpactglobal.org/unaids‐and‐mpact‐are‐extremely‐concerned‐about‐reports‐that‐lgbti‐people‐are‐being‐blamed‐and‐abused‐during‐the‐covid‐19‐outbreak/

[21] De Beck K , Cheng T , Montaner JS , Beyrer C , Elliott R , Sherman S , et al. HIV and the criminalisation of drug use among people who inject drugs: a systematic review. Lancet HIV. 2017;4(8):e357–74. 10.1016/S2352-3018(17)30073-5 PMC6005363 28515014

[22] Dolan K , Wirtz A , Moazen B , Galvani A , Ndeffo‐mbah M , Kinner S , et al. Global burden of HIV, viral hepatitis, and tuberculosis among prisoners and detainees. Lancet. 2016;388(10049):1089–102. 27427453 10.1016/S0140-6736(16)30466-4

[23] Iversen J , Dore G , Catlett B , Cunningham P , Grebely J , Maher L . Association between rapid utilisation of direct hepatitis C antivirals and decline in the prevalence of viremia among people who inject drugs in Australia. J Hepatol. 2019;70(1):33–9. 30367897 10.1016/j.jhep.2018.09.030

[24] Vancouver City News . Dwindling drug supply on DTES drives prices up, leaves users desperate as COVID‐19 closes border [cited 2020 Apr 24]. Available from: https://www.citynews1130.com/2020/03/24/drug‐supply‐bc‐covid‐19‐border/

[25] Dunlop AJ , Lokuge B , Masters D , Sequeira M , Saul P , Dunlop G , et al. Challenges in maintaining treatment services for people who use drugs during the COVID‐19 pandemic. Harm Reduct J. 2020;17:26. 32375887 10.1186/s12954-020-00370-7 PMC7201394

[26] DH News . COVID‐19 highlights the urgency to expand the “safe supply” opioids machine model. 2020 [cited 2020 Apr 24]. Available from: https://dailyhive.com/vancouver/covid‐19‐urgency‐safe‐supply‐opioids‐machine‐model

[27] International Network of People who use Drugs (INPUD) . INPUD online survey on COVID‐19 & people who use drugs (PWUD): Data Report 1. 2020 [cited 2020 Sep 10]. Available from: https://www.inpud.net/sites/default/files/COVID‐19%20Survey%20Data%20Report%2020.pdf

[28] Shannon K , Crago A‐L , Baral SD , Bekker L‐G , Kerrigan D , Decker MR , et al. The global response and unmet actions for HIV and sex Workers. Lancet. 2018;392:698–710. 30037733 10.1016/S0140-6736(18)31439-9 PMC6384122

[29] Deering K , Amin A , Shoveller J , Nesbit A , Garcia‐Moreno C , Duff P , et al. A systematic review of the correlates of violence against sex workers. Am J Public Health. 2014;104(5):e42–54. 10.2105/AJPH.2014.301909 PMC3987574 24625169

[30] Maher L , Dixon TC , Phlong P , Mooney‐Somers J , Stein E , Page K . Conflicting rights: How the prohibition of human trafficking and sexual exploitation infringes the right to health of female sex workers in Phnom Penh, Cambodia. Health and Human Rights. 2015;17(1):102–13. PMC6915836 26204575

[31] Prestage G , Hammoud M , Jin F , Degenhardt L , Bourne A , Maher L . Mental health, drug use and sexual risk behavior among gay and bisexual men? Int J Drug Policy. 2018;55:169–79. 29429865 10.1016/j.drugpo.2018.01.020

[32] Sanchez TH , Zlotorzynsk M , Rai M , Baral SD . Characterizing the impact of COVID‐19 on men who have sex with men across the United States in April, 2020. AIDS Behav. 2020;24:2024‐32. 32350773 10.1007/s10461-020-02894-2 PMC7189633

[33] Hammoud MA , Maher L , Holt M , Degenhardt L , Jin F , Murphy D , et al. Physical distancing due to COVID‐19 disrupts sexual behaviors among gay and bisexual men in Australia: Implications for trends in HIV and other sexually transmissible infections. J Acquir Immune Defic Syndr. 2020;85(3):309–15. 32740374 10.1097/QAI.0000000000002462

[34] Hawks L , Woolhandler S , McCormick D . COVID‐19 in Prisons and Jails in the United States. JAMA Intern Med. 2020;180:1041‐2. 32343355 10.1001/jamainternmed.2020.1856

[35] Wallace M , Hagan L , Curran KG , Williams P , Handanagic S , Bjork A , et al. COVID‐19 in correctional and detention facilities ‐ United States, February‐April 2020. MMWR Morb Mortal Wkly Rep. 2020;69:587–90. 32407300 10.15585/mmwr.mm6919e1

[36] Poteat T , Scheim A , Xavier J , Reisner S , Baral S . Global epidemiology of HIV infection and related syndemics affecting transgender people. J Acquir Immune Defic Syndr. 2016;72 Suppl 3:S210–19. 27429185 10.1097/QAI.0000000000001087 PMC4969059

[37] Poteat T , Wirtz A , Reisner S . Strategies for engaging transgender populations in HIV prevention and care. Curr opin HIV AIDS. 2019;14(5):393–400. 31219887 10.1097/COH.0000000000000563

[38] Wilkinson L , Grimsrud A . The time is now: expedited HIV differentiated service delivery during the COVID‐19 pandemic. J Int AIDS Soc. 2020;23:e25503. 32378345 10.1002/jia2.25503 PMC7203569

[39] United Nations Committee on Economic, Social and Cultural Rights . General comment No. 25 (2020) on Science and economic, social and cultural rights Art. 15.1.b, 15.2, 15.3 and 15.4 [cited 2020 Apr 15]. Available from: https://www.ohchr.org/en/hrbodies/cescr/pages/cescrindex.aspx?eType=EmailBlastContent&eId=6b3d79b7‐aa4c‐4f5e‐acf7‐63266ac002d9

[40] UNAIDS Press Centre . World leaders unite in call for a people’s vaccine against COVID‐19. 2020 [cited 2020 Sep 10]. Available from: https://www.unaids.org/en/resources/presscentre/pressreleaseandstatementarchive/2020/may/20200514_covid19‐vaccine

[41] Strathdee S , Kuo I , El‐Bassel N , Hodder S , Smith L , Springer S . Preventing HIV outbreaks among people who inject drugs in the United States. AIDS. 2020 [cited 2020 Sep 16]. Available from: https://journals.lww.com/aidsonline/Citation/9000/Preventing_HIV_outbreaks_among_people_who_inject.96615.aspx 10.1097/QAD.0000000000002673 PMC7606503 32826391

